# Differences in Trunk Acceleration-Derived Gait Indexes in Stroke Subjects with and without Stroke-Induced Immunosuppression

**DOI:** 10.3390/s24186012

**Published:** 2024-09-17

**Authors:** Luca Martinis, Stefano Filippo Castiglia, Gloria Vaghi, Andrea Morotti, Valentina Grillo, Michele Corrado, Federico Bighiani, Francescantonio Cammarota, Alessandro Antoniazzi, Luca Correale, Giulia Liberali, Elisa Maria Piella, Dante Trabassi, Mariano Serrao, Cristina Tassorelli, Roberto De Icco

**Affiliations:** 1 Department of Brain and Behavioral Sciences, University of Pavia, 27100 Pavia, Italy; luca.martinis@mondino.it (L.M.); gloria.vaghi@mondino.it (G.V.); federico.bighiani@mondino.it (F.B.); elisa.piella@edu.unito.it (E.M.P.);; 2 Movement Analysis Research Section, IRCCS Mondino Foundation, 27100 Pavia, Italy; 3 Department of Medical and Surgical Sciences and Biotechnologies, “Sapienza” University of Rome, 04100 Latina, Italy; stefanofilippo.castiglia@uniroma1.it (S.F.C.);; 4 Movement Analysis Laboratory, Policlinico Italia, 00162 Rome, Italy; 5 Neurology Unit, Department of Clinical and Experimental Sciences, University of Brescia, 25121 Brescia, Italy; 6 Department of Continuity of Care and Frailty, ASST Spedali Civili, 25121 Brescia, Italy; 7 Sports Science Unit, Department of Public Health, Experimental Medicine and Forensic Sciences, University of Pavia, 27100 Pavia, Italygiulia.liberali@unipv.it (G.L.)

**Keywords:** neurological disability, movement analysis, physiotherapy

## Abstract

*Background*: Stroke-induced immunosuppression (SII) represents a negative rehabilitative prognostic factor associated with poor motor performance at discharge from a neurorehabilitation unit (NRB). This study aims to evaluate the association between SII and gait impairment at NRB admission. *Methods*: Forty-six stroke patients (65.4 ± 15.8 years, 28 males) and 42 healthy subjects (HS), matched for age, sex, and gait speed, underwent gait analysis using an inertial measurement unit at the lumbar level. Stroke patients were divided into two groups: (i) the SII group was defined using a neutrophil-to-lymphocyte ratio ≥ 5, and (ii) the immunocompetent (IC) group. Harmonic ratio (HR) and short-term largest Lyapunov’s exponent (sLLE) were calculated as measures of gait symmetry and stability, respectively. *Results*: Out of 46 patients, 14 (30.4%) had SII. HR was higher in HS when compared to SII and IC groups (*p* < 0.01). HR values were lower in SII when compared to IC subjects (*p* < 0.01). sLLE was lower in HS when compared to SII and IC groups in the vertical and medio-lateral planes (*p* ≤ 0.01 for all comparisons). sLLE in the medio-lateral plane was higher in SII when compared to IC subjects (*p* = 0.04). *Conclusions*: SII individuals are characterized by a pronounced asymmetric gait and a more impaired dynamic gait stability. Our findings underline the importance of devising tailored rehabilitation programs in patients with SII. Further studies are needed to assess the long-term outcomes and the role of other clinical features on gait pattern.

## 1. Introduction

Stroke is one of the leading causes of death and severe disability worldwide [1,2]. The outcomes of an acute cerebrovascular event can vary greatly, often resulting in severe motor impairments that significantly hinder the patient’s ability to walk and perform daily activities [3]. Additionally, stroke can have systemic effects on the immune system [4]. During stroke, inflammatory mediators are locally released and detected by the central nervous system, triggering an anti-inflammatory response even in the absence of systemic inflammation [4]. This condition, known as stroke-induced immunosuppression (SII) [5], leads to decreased levels of circulating lymphocytes, natural killer cells, granulocytes, and monocytes [6]. A validated parameter to assess the presence of SII is the neutrophil-to-lymphocyte ratio (NLR) [7,8,9,10], with an NLR ≥ 5 reflecting immunosuppression [11,12,13]. In the acute setting, SII is diagnosed in around 25–30% of people with stroke (PwS) [9]. When evaluated in the intensive care department, SII was associated with stroke volume, neurological disability, rate of infections and pneumonia, and mortality [14,15,16]. By contrast, data regarding its role in the neurorehabilitative field is scarce. A recent study demonstrated a prevalence of SII at neurorehabilitation (NRB) admission of 16% [17]. In this context, PwS bearing SII were characterized by poorer functional, motor, and neurological performances at NRB admission and discharge and also had a higher risk of infectious complications [17].

One of the most disabling consequences of stroke is gait impairment, resulting from strength and sensory deficits, ataxia, and postural instability [18]. Consequently, a significant portion of stroke rehabilitation focuses on improving patients’ walking abilities [3,18]. Independent walking is often viewed as a key indicator of autonomy and overall recovery potential [19]. Current treatment strategies are based on established techniques that include neuromotor training, the use of technological aids, and, more recently, the implementation of innovative therapies such as robotic or virtual reality-based therapies [20]. However, predictors of rehabilitation outcomes are lacking, and many patients do not achieve optimal gait recovery, with severe residual disability [21]. The typical post-stroke gait pattern is characterized by increased gait asymmetry, resulting in imbalance, endurance inefficiency, risk of musculoskeletal injury, loss of bone mass density, difficulties in maintaining a stable gait, and a high risk of falls [22,23,24,25,26,27]. The gait impairment of PwS has been quantified using spatiotemporal gait parameters such as gait speed, step length, swing and stance times [28], as well as synthetic indexes based on spatiotemporal parameters such as the symmetry index [22,29]. These features are not able to completely capture the complexity of post-stroke gait pattern for several reasons: (i) they are directly dependent on gait speed [30], (ii) they do not inform about the quality of body motion translation while walking [28,31,32,33,34], and (iii) they do not describe trunk and pelvic biomechanic alterations [34,35,36,37], although trunk control is a solid predictor of gait recovery [4,38].

Although several measurement tools have been developed to assess gait impairment in PwS, they are designed to capture performance or functional disability rather than a more in-depth quantification of the cardinal signs of gait impairment, such as asymmetry or trunk impairment [39]. Inertial measurement units (IMU) are a viable option for motion analysis in PwS due to the affordability and adaptability to a variety of clinical settings [40]. IMUs can monitor the body’s center of mass while moving the base of support, resulting in effective tools for monitoring dynamic balance during gait [39,41,42]. A single IMU placed at the lower back is sufficient to effectively assess dynamic balance among older adults [43,44] and can accurately identify gait abnormalities in subjects with neurological conditions [45,46,47]. A series of trunk acceleration-derived gait indexes have been proposed to assess dynamic balance based on trunk acceleration patterns [46,48] and to monitor rehabilitation improvement in neurologic conditions [47,49]. Among these parameters, the harmonic ratio (HR), the largest Lyapunov’s exponent for short time series (sLLE) [50,51,52,53], and the log-dimensionless jerk of accelerations (LDLJ) [54,55] proved consistent predictors of trunk and gait rehabilitation in cerebellar ataxia and Parkinson’s disease [46,47,48,49]. In PwS, the harmonic ratio (HR) has been validated for assessing gait asymmetry and compensatory strategies to maintain stability during walking [38,50,51]. Other trunk acceleration-derived indexes, such the largest Lyapunov’s exponent for short time series (sLLE) [52,53] and the log-dimensionless jerk of accelerations (LDLJ) [54,55], have been shown to reflect the compensatory mechanisms that PwS can implement to increase their gait ability, although at the expense of energy demand, gait stability [39,50,56] and gait smoothness [55].

This study aimed to assess whether the presence of SII is associated with the severity of gait impairment at baseline and with the degree of recovery after the rehabilitative intervention. Our working hypothesis was that the presence of SII may be associated with worse trunk acceleration gait behavior when compared to immunocompetent (IC) stroke subjects and healthy subjects (HS) and may negatively influence gait recovery after neurorehabilitation. 

## 2. Materials and Methods

### 2.1. Participants and Study Design

This is a secondary analysis of a previously published study identified SII as a negative predictor of clinical recovery in the neurorehabilitation setting [17]. This prospective and observational study involved PwS admitted for rehabilitation at the Neurorehabilitation Unit (NRB) of the IRCCS Mondino Foundation in Pavia, Italy. A detailed description of the procedures of the parent study was previously published [17]. Briefly, the rehabilitation program lasted between 2 and 8 weeks and consisted of motor rehabilitation (500 min per week across 6 days), along with speech, swallowing, cognitive, and/or occupational therapy tailored to the individual clinical presentation. 

Upon admission at the NRB (T0) of the PwS, we recorded demographic data, pre-stroke functional status using the modified Rankin Scale (mRS), comorbidities, clinical features of stroke, and ongoing therapies. To assess clinical outcomes, we administered the Functional Independence Measure (FIM), the Barthel Index (BI), the National Institutes of Health Stroke Scale (NIHSS), and the Tinetti Balance Score. We also obtained blood samples from the cubital vein for blood cell counts. Based on the output of the test, we stratified PwS in two groups: the SII group formed by subjects with an NLR ≥ 5, and the immunocompetent group (IC group), formed by subjects with NLR < 5. Within two days from admission, subjects underwent the gait analysis, conducted using an IMU (see Section 2.2) to determine gait speed and a series of stability indices derived from trunk acceleration. 

Upon discharge from the NRB (T1), all PwS were reassessed with gait analysis, and the same set of clinical scales recorded at T0. 

The inclusion criteria were: (i) first episode of ischemic stroke or primary spontaneous intracerebral hemorrhage confirmed by neuroimaging; (ii) admission to the NRB within 30 days of the index event; (iii) Functional Ambulation Classification (FAC) ≥ 2 at hospital admission. Exclusion criteria were: (i) age under 18 years; (ii) hospitalization in the NRB for less than 14 days; (iii) medical history of immunodeficiency or immunoproliferative disease; (iv) immunosuppressive or immunomodulating therapy in the 2 years prior to the index event; (v) use of systemic steroids in the 6 months prior to the index event; (vi) Glasgow Coma Scale (GCS) score below 8 at hospital admission; (vii) presence of other major neurological diseases; viii) inability to provide informed consent at hospital admission.

Regarding the HS control group, exclusion criteria were: (i) age under 18 years; (ii) a medical history of neurological diseases, major cardio-vascular comorbidities, diabetes, alcohol abuse, musculoskeletal diseases impairing gait, immunodeficiency or immunoproliferative disease; (iii) inability to provide informed consent at hospital admission.

The local Ethics Committee approved the study (2022-3.11/58), and it was registered on www.clinicaltrials.gov (NCT05889169). The procedures used in this study adhere to the principles of the Declaration of Helsinki. The dataset generated and/or analyzed during the current study is available in the Zenodo repository (https://doi.org/10.5281/zenodo.10057752 (12 September 2024)). The dataset is available from the corresponding author upon reasonable request. Informed written consent was obtained from all subjects before enrollment.

### 2.2. Gait Data Acquisition 

Gait parameters were measured by means of a single IMU (G-WALK, BTS, Milan, Italy) placed at the L5 level. The sensor incorporates a triaxial accelerometer (16 bits/axis), a triaxial magnetometer (13 bits), and a triaxial gyroscope (16 bits/axis). Linear trunk accelerations and angular velocities in the anterior–posterior (AP), mediolateral (ML), and vertical (V) directions were recorded at 100 Hz using the “Walk+” protocol of the G-STUDIO software (version 3.5.25.0) (G-STUDIO, BTS, Milan, Italy). The software was also used to estimate spatiotemporal gait parameters, including gait speed, stance, and swing phases, as well as double and single support subphases. Both PwS and HS were instructed to walk at their own pace along a 30 m long and 3 m wide corridor. Since our goal was to investigate natural and spontaneous locomotion, we provided participants with only general and qualitative instructions, allowing them to choose their own gait speed without any external sensory input. HS were also instructed to walk at a slower gait speed to increase the number of possible combinations for the matching procedure. We specifically instructed the subjects to begin walking immediately after the calibration process in the “Walk+” protocol, maintain a consistent gait, and stop at the end of the path. During the gait acquisition procedure, a physiotherapist walked behind and slightly lateral to the patient in order to intervene quickly in the event of an adverse event. Five participants required assistance and physical support while walking by a caregiver or using a walking device. No adverse events occurred during the experiment.

To calculate the trunk acceleration-derived gait indexes, gait trials with at least 20 consecutive accurately recorded strides were included in the analysis [46,47,51,57,58]. To ensure a steady-state walking assessment, we removed the first and last two strides of each 30-m walk. To identify initial contacts (positive peaks occurring between the zero-crossings), the vertical acceleration signal was first detrended and then low-pass filtered using an FIR filter set at 3.2 Hz [59,60,61,62]. The resulting signal was numerically integrated and differentiated using a Gaussian continuous wavelet transformation (scale 9, gauss 1). 

#### 2.2.1. Harmonic Ratio (HR) Calculation

The harmonic ratio (HR) [47,48,49,50,63] is an indicator of gait symmetry in PwS, in the antero-posterior (HR_AP_), medio-lateral (HR_ML_), and vertical (HR_V_) directions. Twenty harmonics were computed for each subject based on stride time. A discrete Fourier transformation was adopted to convert the trunk accelerations of each stride into single sinusoidal waveforms. HR_AP_ and HR_V_ were calculated by dividing the sum of the first ten even harmonics by the sum of the first ten odd harmonics. HR_ML_ was calculated by adding the amplitudes of the odd harmonics and dividing them by the amplitudes of the even harmonics [64,65,66,67,68,69]. To eliminate noise signals, a high-pass filter with a cutoff of 20 Hz was used [70]. HRs were calculated for each stride and averaged over a steady walk to obtain a mean HR as follows:

$${HR}_{AP,\;V\;}=\frac{{\sum_{i}A}_{i*2}}{\sum_{i}A_{i*2-1}}$$

$${HR}_{ML\;}=\frac{\sum_{i}A_{i*2-1}}{{\sum_{i}A}_{i*2}}$$

where $A_{i\;}$ indicates the amplitude of the first 20 even harmonics, and A2i–1 represents the amplitude of the first 20 odd harmonics. 

#### 2.2.2. Short-Term Largest Lyapunov Exponent (sLLE) Calculation

The short-term largest Lyapunov’s exponent (sLLE) [49,52,53,64,65] represents a measure to quantify gait stability. For each subject, with a set number of n = 20 strides, accelerations were divided into segments based on predefined initial contacts, where each segment corresponded to a gait stride. The data were then time-normalized to produce 100 data points for each stride [71]. To preserve spatiotemporal fluctuations and nonlinearities, the acceleration signals were not filtered [72]. The recorded one-dimensional time-series data was transformed into a multidimensional state space by juxtaposing the original data with delayed copies. The time delay (*τ*) was calculated using the first minimum of the Average Mutual Information (AMI) function, exploring a range from 7 to 18 samples. The dimensions of the reconstructed state space were determined using the False Nearest Neighbor (FNN) method, with a maximum embedding dimension of 10 [46,49,65,71]. The sLLE for each stride and acceleration direction was calculated using Rosenstein’s algorithm for short time series, which was implemented using the “lyap_r” function from the “nolds” library in Python.

#### 2.2.3. Log-Dimensionless Jerk of Accelerations (LDLJ) Calculation 

The log-dimensionless jerk of acceleration signals (LDLJ) [55,66] reflects the smoothness of acceleration. The acceleration data were first filtered using a low-pass Butterworth filter with a cutoff frequency of 20 Hz and a sampling frequency of 100 Hz to remove high-frequency noise. Then, the LDLJ was computed according to the formula:

$$\lambda_{L}^{a}\left(a\right)≜-\ln\left(\frac{\left(t_{2}-t_{1}\right)}{a_{\mathrm{peak}}^{2}}\int_{t_{1}}^{t_{2}}\left.|\frac{d^{2}}{dt^{2}}a\left(t\right)\right.{|}_{2}^{2}\right)$$

$$a_{\mathrm{peak}}≜\underset{t\in \left[t_{1},t_{2}\right]}{\max}|a\left(t\right){|}_{2}$$

where *a*(*t*) denotes the movement acceleration in the time domain, a normalization factor based on the peak of the acceleration profile $a_{peak}$, and $t_{1}$,$\;t_{2}$ represent the start and stop times of the movement, respectively. 

#### 2.2.4. Symmetry Indexes Calculation

The symmetry index (SI) [22] was calculated considering the temporal gait parameters [26] stance time, swing time, double support, and single support times according to the formula [22]:

$$SI=\left[\frac{{(V}_{paretic}-V_{non\;paretic})}{0.5*(V_{paretic}+V_{non\;paretic})}\right]*100$$

where *V* represents the mean values of the gait parameters over the 20 recorded strides. 

### 2.3. Statistical Analysis

The primary objective of this study was to assess differences in the HR values across the three study groups at T0. Sample size calculation was performed with the G*Power software (ver. 3.1.9.6). We estimated a sample size of at least 78 PwS for a F tests with three groups, assuming an effect size of at least 0.46, at a significance level of 0.05 and a desired power of 0.80 [39,48]. The Shapiro-Wilk and Levene’s tests were used to assess the distributions’ normality and homogeneity, respectively. The chi-squared test for categorical variables and the Mann–Whitney test for continuous variables were used to compare clinical variables between the IC and SII groups. To compare trunk acceleration-derived gait indexes between the HS, IC, and SII groups, a Kruskal–Wallis test with Dunn’s post hoc analysis and Holm’s correction was implemented. Eta-squared was calculated as the effect size. 

A non-parametric repeated measures analysis was performed using time (within subjects, two levels: T0 and T1) and group (between subjects, two levels: SII vs. IC) to assess the association between the presence of SII and the modifications between admission (T0) and discharge (T1). The repeated measure analysis was performed only for those gait variables that resulted in a significant difference between PwS and HS at T0. The timeXgroup interaction was investigated to identify differences in improvement rates between T0 and T1 between the SII and IC groups, and additional post hoc analyses were performed only in case of a significant interaction. Age, sex, and time from stroke onset to admission into NRB, stroke volume [73] as well as the percentage improvements in Barthel Index and NIHSS scores between T0 and T1 were included as covariates in the model. 

Statistical analyses were conducted using JASP vers. 0.18.3 software, and the “nparLD” package in R (R: A language and environment for statistical computing”, R Foundation for Statistical Computing, Vienna, Austria). The significance level was set at 95% and power at 80% for all the analyses. 

## 3. Results

### 3.1. Study Population

This study included 46 PwS (39.1% female, 65.4 ± 15.8 years). Ischemic stroke was diagnosed in 93.5% of participants. Compared to the parent study, 50 PwS were excluded from the analysis because of a FAC < 2 at T0. All 46 subjects presented moderate hemiparesis and were admitted to the NRB after an average of 6.9 ± 3.8 days from stroke onset. SII was identified in 14 (30.4%) of them. Table 1 describes the clinical and demographic features of the included PwS upon admission to the NRB. Aside from NLR values, differences were found between the SII and IC groups regarding the presence of dysphagia, which was present in 42.9% of PwS with SII compared to 9.4% in the IC group (*p* = 0.01), as well as the NIHSS score (*p* = 0.04), which was higher in the SII group, and the BI score (*p* = 0.01), which was lower in the SII group (Table 1). The average gait speed of PwS was 0.69 ± 0.24 m/s.

After the matching procedure, 42 HS were included, 16 females (38.1%), with an average age of 62.1 ± 8.7 years. The average gait speed was 0.78 ± 0.19 m/s. At T0, there were no differences in age (*p* = 0.07), sex (*p* = 0.94), and gait speed (*p* = 0.11) between PwS and HS (Table 2).

### 3.2. Modification of Clinical Scales after Rehabilitation (T1)

At T1, FIM score improved in the overall study population (T0 = 85.4 ± 19.7; T1 = 113.0 ± 13.1, factor TIME: *p* < 0.01). The rate of improvement did not differ between the SII and IC groups (interaction TIMExGROUP: *p* = 0.44). After rehabilitation, functional independence was comparable between SII (110.0 ± 14.5) and IC (109.0 ± 12.6) groups (factor GROUP: *p* = 0.86).

At T1, NIHSS score improved in the overall study population (T0 = 5.6 ± 3.3; T1 = 3.1 ± 2.8, factor TIME: *p* < 0.01). The rate of improvement did not differ between SII and IC groups (interaction TIMExGROUP: *p* = 0.75). After rehabilitation, neurological disability was comparable between SII (3.5 ± 3.5) and IC (2.9 ± 2.5) groups (factor GROUP: *p* = 0.56).

At T1, BI score improved in the overall study population (T0 = 54.6 ± 20.4; T1 = 89.7 ± 10.7, factor TIME: *p* < 0.01). The rate of improvement did not differ between SII and IC groups (interaction TIMExGROUP: *p* = 0.14). After rehabilitation, performance in the activities of daily living was comparable between SII (87.9 ± 14.0) and IC (90.5 ± 9.0) groups (factor GROUP: *p* = 0.09).

At T1, Tinetti score improved in the overall study population (T0 = 17.4 ± 8.0; T1 = 24.7 ± 3.9, factor TIME: *p* < 0.01). The rate of improvement did not differ between SII and IC groups (interaction TIMExGROUP: *p* = 0.66). After rehabilitation, motor performance was comparable between SII (23.9 ± 4.7) and IC (25.1 ± 3.5) groups (factor GROUP: *p* = 0.35).

### 3.3. Baseline (T0) Trunk Acceleration-Derived Gait Indexes

At T0, gait speed did not differ among SII group (0.66 ± 0.26 m/s), IC group (0.72 ± 0.21 m/s), and HS (0.78 ± 0.19 m/s) (*p* = 0.11).

**Figure 1:**
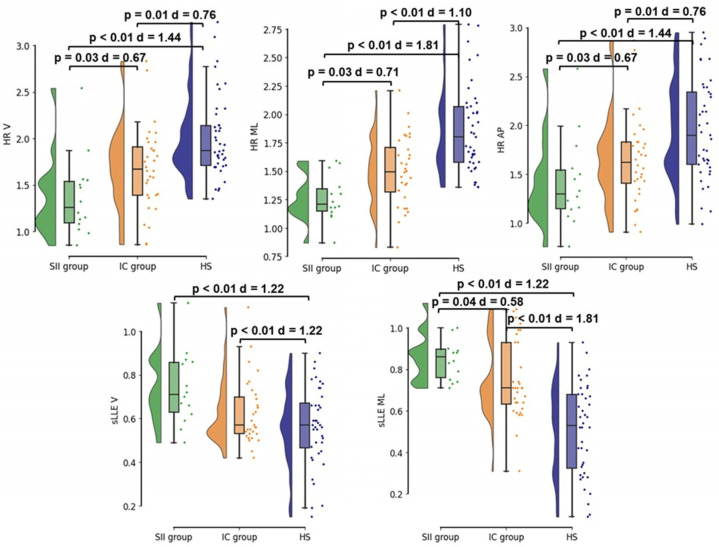
Post hoc analysis for trunk acceleration-derived gait indexes at T0 among the three study groups.

Legend: SII group, stroke-induced immunosuppression group (green); IC group, immunocompetent group (orange); HS, age and gait speed-matched healthy subjects (blue); HR, harmonic ratio; sLLE, short–term Lyapunov’s exponent; V, vertical direction of the acceleration signal; ML, medio-lateral direction of the acceleration signal; AP, antero-posterior direction of the acceleration signal.

HR was different among SII group, IC group, and HS in the vertical (*p* < 0.01), medio-lateral (*p* < 0.01), and antero-posterior (*p* < 0.01) planes *(*Table 2). At post hoc analysis, HR_V_ was higher in HS (2.01 ± 0.47) when compared to SII group (1.36 ± 0.45, *p* < 0.01) and IC group (1.68 ± 0.45, *p* = 0.01); in addition, HR_V_ was lower in SII group when compared to IC group (*p* = 0.03) (Figure 1). HR_ML_ was higher in HS (1.87 ± 0.39) when compared to SII group (1.24 ± 0.19, *p* < 0.01) and IC group (1.50 ± 0.30, *p* < 0.01); in addition, HR_ML_ was lower in SII group when compared to IC group (*p* = 0.03) (Figure 1). HR_AP_ was higher in HS (1.95 ± 0.51) when compared to SII group (1.41 ± 0.48, *p* < 0.01) and IC group (1.64 ± 0.44, *p* = 0.01); in addition, HR_AP_ was lower in SII group when compared to IC group (*p* = 0.03) (Figure 1).

sLLE was different among SII group, IC group, and HS in the vertical (*p* = 0.01), and medio-lateral (*p* < 0.01) planes, but not in the antero-posterior plane (*p* < 0.14) (Table 2). At post hoc analysis, sLLE_V_ was lower in HS (0.56 ± 0.18) when compared to SII group (0.75 ± 0.17, *p* < 0.01) and IC group (0.63 ± 0.15, 0 < 0.01); sLLE_V_ did not differ between SII and IC groups (*p* > 0.05) (Figure 1). sLLE_ML_ was lower in HS (0.51 ± 0.22) when compared to the SII group (0.87 ± 0.12, *p* < 0.01) and the IC group (0.75 ± 0.19, *p* < 0.01); in addition, sLLE_ML_ was higher in the SII group when compared to the IC group (*p* = 0.04) (Figure 1).

LDLJ in all of the three planes, SI stance, SI swing, SI double support, and SI single support did not differ among the three study groups (*p* > 0.05 for all comparisons) (Table 2).

### 3.4. Modification of Trunk-Acceleration-Derived Gait Indexes after Rehabilitation (T1)

As previously described, only trunk acceleration-derived indexes that were different between PwS and HS at T0 were analyzed for modifications after the rehabilitative treatment.

At T1, we observed a HR improvement in the overall clinical population, specifically in HR_V_ (T0: 1.58 ± 0.46 vs. T1: 1.93 ± 0.62; TIME: *p* = 0.02), in HR_ML_ (T0: 1.43 ± 0.30 vs. T1: 1.70 ± 0.49; TIME: *p* < 0.01), and in HR_AP_ (T0: 1.58 ± 0.46 vs. T1: 1.86 ± 0.52; TIME: *p* < 0.03) (Table 3). Also, sLLE improved in the overall population for both sLLEv (T0: 0.66 ± 0.16 vs. T1: 0.59 ± 0.18; TIME: *p* = 0.03), and sLLE_ML_ (T0: 0.78 ± 0.18 vs. T1: 0.71 ± 0.17; TIME: *p* < 0.01) (Table 3) as well as gait speed (T0: 0.70 ± 0.23 vs. T1: 0.90 ± 0.27; TIME: *p* < 0.01) (Table 3). Between T0 and T1, gait pattern improved in both SII and IC subjects, with the degree of improvement being comparable between study groups (interaction TIMExGROUP: *p* > 0.05 for all comparisons). At the end of rehabilitation, SII group was still characterized by a more impaired gait pattern compared to IC participants. This is suggested by a significant factor GROUP (*p* < 0.05 for all comparisons) for all the previously described trunk acceleration-derived gait indexes (Table 3).

## 4. Discussion

In the present study, we evaluated whether the presence of SII in PwS is associated with a worse gait pattern compared to immunocompetent PwS and to healthy controls. We also assessed whether the presence of SII may negatively influence gait recovery after rehabilitation.

Our results may be summarized as follows:

- At NRB admission, SII subjects were characterized by a more severe gait pattern when compared to the IC and HS groups. Specifically, SII participants showed lower HR values, which are consistent with a more asymmetric gait pattern, and higher sLLE values, suggesting a more impaired gait stability.
- The rehabilitative intervention ameliorated the gait behavior in both IC and SII stroke subjects with improvements observed in the HRs in the three spatial directions, sLLE in the vertical and medio-lateral panels, and in gait speed. These findings are consistent with an improvement in trunk symmetry and dynamic stability during gait, as well as overall gait performance.
- Although the rehabilitative intervention improved gait pattern to a similar extent in both groups, gait of SII patients was more compromised at both NRB admission and discharge when compared with IC group. Thus, SII qualifies as a negative prognostic factor for gait rehabilitation, although its presence did not hamper a certain degree of improvement.

These results are consistent with existing literature reporting lower HR values in subjects with sub-acute stroke [50,63,67,74], reflecting lower symmetry of trunk acceleration pattern compared with healthy participants, as well as with studies reporting abnormalities in local dynamic stability during gait in stroke individuals [53,64]. Although this is a secondary analysis of a previously published study, this is the first report regarding the association between SII and worse HR and sLLE values at NRB admission. Furthermore, we found HRs and sLLE to improve following rehabilitation, regardless of age, sex, time from stroke onset to admission into NRB, stroke volume, as well as the percentage improvements in Barthel Index and NIHSS scores between T0 and T1. This suggests responsiveness to inpatient neurorehabilitation of symmetry and dynamic stability of the trunk during gait in PwS [64,75]. While both groups improved in gait function after rehabilitation, SII subjects generally had worse dynamic trunk symmetry, stability, and gait performance. By contrast, the degree of improvement was comparable regardless of the immunocompetence status. It is noteworthy that in our cohort, NIHSS and BI scores were comparable between SII and IC groups at the end of rehabilitation, while the trunk acceleration-derived gait indexes were not. This observation may suggest a clinical role of gait analysis to monitor stroke patients, as it appears more sensitive when compared to clinical scales to detect a difference at least in gait performance.

These findings further expand on our previous results describing how SII patients were characterized by higher disability levels at neurorehabilitation admission and discharge [9]. Indeed, Vaghi et al. reported more compromised disability domains in the SII group in the NRB setting, with similar rates of improvement between IC and SII groups in functional independence, activities of daily living, and motor performance [17]. These results highlight the importance of accounting for immunological status for tailoring rehabilitation strategies to maximize dynamic trunk and gait recovery in stroke subjects.

Notably, we did not find significant differences between PwS and HS in symmetry indexes based on spatio-temporal gait parameters, nor in gait smoothness, as calculated through the LDLJ of trunk accelerations, regardless of gait speed. In this study, we quantified symmetry indexes based on temporal phases of the gait cycle. Although these measures are highly responsive to physical therapy interventions, they may not differentiate the mechanisms because they scale to gait speed [30] in stroke subjects. In this study, HS were also asked to walk at a slower gait speed than their preferred one in order to enhance the effectiveness of the gait speed matching procedure. In this way, the differences in temporal asymmetry between the groups may have been reduced because of the increase of temporal asymmetry in healthy participants due to the slower walking pace [76]. The LDLJ has also been described as scaling to acceleration fluctuations due to the variability in walking speed. Thus, the lack of significant differences at admission between PwS and HS may still be attributed to the effectiveness of the gait speed matching procedure, as proved by other studies on subjects suffering from different neurologic conditions [46,47,48,49]. 

From a pathophysiological perspective, several mechanisms may explain the association between SII and gait impairment. SII and central nervous system damage are linked to a dysfunction in the sympathetic autonomic response and to an uncontrolled release of cytokines and immune mediators [77]. The sympathetic reflex is essential during physically stressful tasks, including reaching an upright standing position and performing gait after a stroke [78]. These processes may also influence the cellular energy metabolism at the cardiovascular level, further aggravating motor and endurance performances in PwS and SII [79]. Furthermore, the high infectious risk may hamper and limit gait recovery during the early phases after stroke. An alternative hypothesis is that SII might only be an epiphenomenon due to a spurious statistical association with the recorded gait pattern. Indeed, we described how PwS and SII are characterized by a pronounced motor impairment and neurological deficit. In this scenario, the severe gait impairment could represent only a byproduct of the underlying motor impairment.

The implications of these findings are relevant for the rehabilitation of stroke patients, particularly for those with immunosuppression. The observed more severe gait abnormalities in the SII group highlight the need for specifically tailored rehabilitation programs. As a future perspective, it would be helpful to study whether the introduction of interventions aimed at reducing systemic inflammation and improving immune function may enhance motor recovery and overall functional outcomes. Additionally, gait training programs should consider incorporating trunk stability and balance exercises to mitigate the risk of falls and improve gait efficiency in SII patients. Longitudinal studies are also needed to determine the long-term burden of SII on motor recovery and functional independence in stroke subjects. Understanding the interplay between the immune system and motor function post-stroke will be crucial in developing comprehensive rehabilitation approaches that optimize recovery and quality of life after stroke.

Several limitations of the present study should be acknowledged. Our follow-up period was relatively short (approximately 45 days), preventing definitive conclusions about long-term outcome. As described, this is a secondary analysis of a previously published study and, although a sample size calculation was performed for the primary outcome, our population dimension might not ensure general applicability of our findings. This is also due to the numerosity imbalance between the IC and SII groups. Indeed, SII was identified in 14 (30.4%) out of 46 stroke patients, limiting the overall generalizability of our results. It is worth noting that the prevalence of SII in neurorehabilitation (15.6%) [17] was previously undocumented, complicating evidence-based calculations. Additionally, without a universally accepted definition of SII, other parameters beyond NLR might better indicate this condition. It is also worth noting that patients with SII were characterized by a more severe stroke phenotype at NRB admission, as demonstrated by a higher NIHSS score and a lower BI score. For this reason, we cannot completely disentangle if this subgroup described worse gait pattern is directly related to the presence of SII or to the pronounced severity of the neurological clinical pattern. Regarding HS, we excluded subjects with neurological diseases, major cardiovascular disorders, diabetes or alcohol abuse, and musculoskeletal disorders affecting gait, but we are lacking data on possible other comorbidities, namely hypertension, hypercholesterolemia, or smoking habit. Lastly, we included patients with both ischemic and hemorrhagic stroke, which may have different rehabilitative outcomes.

## 5. Conclusions

Understanding the interplay between the immune system and motor function post-stroke will be crucial in developing comprehensive rehabilitation approaches to optimize recovery.

The differences observed between healthy participants and stroke patients, as well as between stroke subgroups based on the presence of SII, indicate that trunk symmetry and dynamic stability during gait may represent useful markers for assessing post-stroke recovery. The results of this study also highlight the importance of considering gait quality, in addition to gait speed, in the rehabilitation of stroke patients. The clinical implications underlie the importance of devising tailored rehabilitation programs in patients with SII. 

Further research and longitudinal studies are needed to explore the underlying mechanisms linking SII to impaired gait parameters, long-term outcomes, and to investigate potential therapeutic strategies.

## Figures and Tables

**Table 1:** Clinical and demographic features of the study population at NRB admission.

		Total (n = 46)	IC Group (n = 32)	SII Group (n = 14)	*p*-Value
Sex (n (%))	F	18 (39.13%)	12 (37.50%)	6 (42.86%)	0.73
	M	28 (60.87%)	20 (62.50%)	8 (57.14%)	
Stroke type (n (%))	Ischemic	43 (93.48%)	31 (96.86%)	12 (85.71%)	0.16
	Hemorrhagic	3 (6.52%)	1 (3.12%)	2 (15.38%)	
Side of lesion (n (%))	Left	18 (39.13%)	13 (28.26%)	5 (10.87%)	0.75
	Right	28 (60.87%)	19 (41.30%)	9 (19.56%)	
Hemorrhagic transformation in ischemic stroke (n (%))	Yes	4 (9.30%)	3 (9.67%)	1 (8.33%)	1.00
	No	39 (90.70%)	28 (90.32%)	11 (91.67%)	
Stroke volume (cm^3^/mL)		16.10 (35.85)	15.76 (32.94)	16.95 (42.22)	0.32
Number of lesions (n (%))	Single	30 (65.22%)	22 (68.75%)	8 (57.14%)	0.45
	Multiple	16 (34.78%)	10 (31.25%)	6 (42.86%)	
Aphasia (n (%))	Yes	12 (26.09%)	6 (18.75%)	6 (42.86%)	0.09
	No	34 (79.13%)	26 (81.25%)	8 (57.14%)	
Dysphagia (n (%))	Yes	9 (19.57%)	3 (9.38%)	6 (42.86%)	0.01
	No	37 (80.43%)	29 (90.63%)	8 (57.14%)	
Cognitive impairment (n (%))	Yes	10 (21.74%)	8 (25%)	2 (14.29%)	0.42
	No	36 (78.26%)	24 (75%)	12 (85.71%)	
Hypertension (n (%))	Yes	38 (82.61%)	25 (78.12%)	13 (92.86%)	0.22
	No	8 (17.39%)	7 (21.87%)	1 (7.14%)	
Hypercholesterolemia (n (%))	Yes	27 (58.70%)	14 (43.75%)	9 (64.29%)	0.61
	No	19 (42.30%)	18 (56.25%)	5 (64.29%)	
Alcohol abuse (n (%))	Yes	16 (34.78%)	12 (37.50%)	4 (28.57%)	0.84
	No	29 (65.21%)	20 (65.5%)	10 (71.42%)	
Diabetes (n (%))	Yes	13 (28.26%)	11 (34.37%)	2 (14.29%)	0.16
	No	33 (71.74%)	21 (65.62%)	12 (85.71%)	
Smoking (n (%))	Active	16 (34.78%)	12 (37.50%)	4 (28.57%)	0.69
	Former	10 (21.74%)	7 (21.87%)	3 (21.43%)	
	Never	20 (43.48%)	13 (40.62%)	7 (50.00%)	
NLR		3.31 (1.90)	2.70 (0.98)	6.68 (2.28)	0.01
Time from stroke onset to NRB admission (days)		6.89 (3.85)	7.53 (4.28)	5.43 (2.06)	0.09
Length of NRB hospitalization (days)		44.65 (14.39)	45.44 (13.90)	45.86 (18.51)	0.97
NIHSS		5.56 (3.29)	5.15 (2.47)	7.86 (5.96)	0.04
BI		54.56 (20.43)	57.05 (19.92)	40.71 (18.80)	0.02
FIM		85.41 (19.69)	86.61 (19.39)	77.33 (23.23)	0.33
Tinetti		17.40 (8.03)	18.0 (7.57)	15.90 (9.12)	0.54

Legend: IC group, immunocompetent stroke patients at admission; SII group, stroke patients with stroke-induced immunosuppression at admission; F, females; M, males; NLR, neutrophil-to-lymphocyte ratio; NRB, neurorehabilitation unit; NIHSS, National Institutes of Health Stroke Scale; BI, Barthel Index; FIM, Functional Independence measure.

**Table 2:** Differences at T0 between the three experimental groups.

		IC group (n = 32)	SII group (n = 14)	HS (n = 42)	H	*p*-Value	η²
		Mean (SD)	Mean (SD)	Mean (SD)			
Age		62.9 (15.9)	71.1 (14.2)	62.1 (8.7)	6.31	**0.07**	0.06
Sex	F M	12 (37.50%) 20 (62.50%)	6 (42.86%) 8 (57.14%)	16 (39.02%) 24 (60.98%)	0.12 *	0.94	
Gait speed		0.72 (0.21)	0.66 (0.26)	0.78 (0.19)	4.48	0.11	0.05
HR V		1.68 (0.45)	1.36 (0.45)	2.01 (0.47)	21.58	<0.01	0.23
HR ML		1.50 (0.30)	1.24 (0.19)	1.87 (0.39)	32.04	<0.01	0.34
HR AP		1.64 (0.44)	1.41 (0.48)	1.95 (0.51)	14.40	<0.01	0.16
sLLE V		0.63 (0.15)	0.75 (0.17)	0.56 (0.18)	8.69	0.01	0.12
sLLE ML		0.75 (0.19)	0.87 (0.12)	0.51 (0.22)	31.41	<0.01	0.35
sLLE AP		0.60 (0.14)	0.71 (0.23)	0.54 (0.23)	3.92	0.14	0.07
LDLJa V		−4.95 (0.31)	−5.03 (0.37)	−4.95 (0.31)	0.98	0.61	0.06
LDLJa ML		−5.44 (0.33)	−5.40 (0.39)	−5.54 (0.27)	3.70	0.16	0.03
LDLJa AP		−5.05 (0.45)	−4.94 (0.24)	−4.98 (0.34)	1.20	0.55	0.01
SI_stance (%)		7.40 (9.08)	7.82 (8.79)	3.48 (2.90)	3.22	0.20	0.08
SI_swing (%)		9.44 (11.16)	12.22 (13.13)	5.82 (4.75)	3.29	0.19	0.07
SI_double support (%)		16.96 (19.50)	24.53 (18.18)	17.94 (15.06)	2.78	0.25	0.03
SI_single support (%)		10.60 (13.18)	12.10 (12.98)	5.60 (4.91)	2.95	0.23	0.07

Legend: IC group, immunocompetent stroke survivors at admission; SII group, stroke patients with stroke-induced immunosuppression at admission; HS, age- and gait speed-matched healthy subjects; HR, harmonic ratio; sLLE, short-term Lyapunov’s exponent; LDLJa, log—dimensionless jerk score; SI, symmetry index. H, Kruskal–Wallis H test; η² Eta-squared. * Chi–squared test.

**Table 3:** Effect of rehabilitation in stroke patients—repeated measures comparisons.

	T0 Mean (SD)	T1 Mean (SD)	IC group		SII group		Factor (*p*-Values)		
			T0 Mean (SD)	T1 Mean (SD)	T0 (Mean SD)	T1 Mean (SD)	Time (T0 vs. T1)	Group (IC vs. SII)	TimeXGroup Interaction
HR_V_	1.58 (0.46)	1.93 (0.62)	1.68 (0.45)	2.04 (0.64)	1.36 (0.45)	1.67 (0.49)	0.02	<0.01	0.59
HR_ML_	1.43 (0.30)	1.70 (0.49)	1.50 (0.30)	1.79 (0.51)	1.24 (0.19)	1.47 (0.38)	<0.01	<0.01	0.88
HR_AP_	1.58 (0.46)	1.86 (0.52)	1.64 (0.44)	1.91 (0.53)	1.41 (0.48)	1.75 (0.50)	0.03	<0.01	0.35
sLLE_V_	0.66 (0.16)	0.59 (0.18)	0.63 (0.15)	0.57 (0.17)	0.75 (0.17)	0.62 (0.19)	0.02	0.02	0.55
sLLE_ML_	0.78 (0.18)	0.71 (0.17)	0.75 (0.19)	0.68 (0.16)	0.87 (0.12)	0.78 (0.18)	<0.01	0.02	0.37
Gait speed (m/s)	0.70 (0.23)	0.90 (0.27)	0.72 (0.21)	0.90 (0.26)	0.66 (0.26)	0.88 (0.29)	<0.01	<0.01	0.39

ML, medio-lateral direction of the acceleration signal; AP, antero-posterior direction of the acceleration signal; T0, assessment time at admission; T1, assessment time at discharge; IC, immunocompetent stroke patients; SII, patients with stroke-induced immunosuppression.

## Data Availability

The procedures used in this study adhere to the tenets of the Declaration of Helsinki. The dataset generated and/or analyzed during the current study is available in the Zenodo repository (https://doi.org/10.5281/zenodo.10057752). The dataset is available from the corresponding author upon reasonable request.
